# Sequence analysis for detection of first-line drug resistance in *Mycobacterium tuberculosis* strains from a high-incidence setting

**DOI:** 10.1186/1471-2180-12-90

**Published:** 2012-05-30

**Authors:** Silke Feuerriegel, Barbara Oberhauser, Abu Garawani George, Foday Dafae, Elvira Richter, Sabine Rüsch-Gerdes, Stefan Niemann

**Affiliations:** 1 Molecular Mycobacteriology, Research Center Borstel, Borstel, Germany; 2 German Leprosy and TB Relief Association, Würzburg, Germany; 3 National Leprosy/TB Reference Laboratory, Freetown, Sierra Leone; 4 Manager of the National Leprosy and Tuberculosis Programme (NLTP), Ministry of Health and Sanitation, Freetown, Sierra Leone; 5 National Reference Center for Mycobacteria, Research Center Borstel, Borstel, Germany

## Abstract

**Background:**

Drug resistance displays a problem for the therapy of *Mycobacterium tuberculosis* infections. For molecular resistance testing, it is essential to have precise knowledge on genomic variations involved in resistance development. However, data from high-incidence settings are only sparely available. Therefore we performed a systematic approach and analyzed a total of 97 *M. tuberculosis* strains from previously treated patients in Sierra Leone for mutations in *kat*G, *rpo*B, *rrs*, *rps*L, *gid*B, *emb*B, *pnc*A and where applicable in *inh*A and *ahp*C. Of the strains investigated 50 were either mono- or poly-resistant to isoniazid, rifampin, streptomycin, ethambutol and pyrazinamide or MDR and 47 fully susceptible strains served as controls.

**Results:**

The majority of isoniazid and rifampin resistant strains had mutations in *kat*G315 (71.9%) and *rpo*B531 (50%). However, *rpo*B mutations in codons 511, 516 and 533 were also detected in five rifampin susceptible strains. MIC determinations revealed low-level rifampin resistance for those strains. Thus, the sensitivity and specificity of sequencing of *kat*G for detection of drug resistance were 86.7% and 100% and for sequencing of *rpo*B 100% and 93.8%, respectively.

Strikingly, none of the streptomycin resistant strains had mutations in *rrs*, but 47.5% harboured mutations in *rps*L. Further changes were detected in *gid*B. Among ethambutol resistant strains 46.7% had mutations at *emb*B306. Pyrazinamide resistant strains displayed a variety of mutations throughout *pnc*A. The specificities of sequencing of *rps*L, *emb*B and *pnc*A for resistance detection were high (96-100%), whereas sensitivities were lower (48.8%, 73.3%, 70%).

**Conclusions:**

Our study reveals a good correlation between data from molecular and phenotypic resistance testing in this high-incidence setting. However, the fact that particular mutations in *rpo*B are not linked to high-level resistance is challenging and demonstrates that careful interpretation of molecular resistance assays is mandatory. In addition, certain variations, especially in *gid*B, appear to be phylogenetically informative polymorphisms rather than markers for drug resistance.

## Background

With more than 9 million new tuberculosis (TB) cases and about 1.7 million deaths in 2009 [1] TB remains one of the most serious infectious diseases worldwide. Treatment and control of TB is further complicated by the emergence of drug resistant and even multi drug resistant (MDR) strains [resistance to at least isoniazid (INH) and rifampin (RIF)] [2]. Among high-incidence settings, Sub-Saharan Africa is eminently affected with two million new TB cases per year [3]. This study focuses on Sierra Leone, a high burden country with an annual TB incidence rate of 574 per 100.000 people and an annual mortality rate of 149 per 100.000 people. Treatment options are further hampered by the fact that 23% among previously treated TB patients in Sierra Leone suffer from an MDR *M. tuberculosis* strain [4].

Rapid detection of resistance is the key task to ensure an effective treatment of patients and also to avoid further spread of resistant *M. tuberculosis* strains. Molecular assays that detect the genetic variants that mediate resistance constitute a rapid alternative to conventional drug susceptibility testing (DST) and may even be performed directly on clinical specimens without culture [5,6]. Therefore it is essential to elucidate the genetic basis of clinical resistance and to correlate phenotypic and molecular resistance data.

Resistance to INH is predominantly mediated by one mutation in the *kat*G gene at codon 315 which results in the complete or partial loss of catalase-peroxidase activity [7]. Further mutations in the promoter regions of *inh*A [8] and *ahp*C [9,10] are associated with INH resistance. Mutations responsible for RIF resistance are primarily located in the so-called rifampin resistance determining region (RRDR; codon 507–533 according to *E. coli* numbering system) of the *rpo*B gene which encodes the beta subunit of the RNA polymerase [11]. Resistance to streptomycin (SM) is mediated by mutations in different genes. Polymorphisms in *rrs* and *rps*L, coding for 16 S rRNA and the ribosomal protein S12, respectively, are mainly responsible for high-level resistance [12]. Recently, the *gid*B gene, which encodes a 7-methylguanosine methyltransferase specific for 16 S rRNA, has additionally been associated with SM resistance [13]. Resistance to ethambutol (EMB) is primarily mediated by mutations in the *emb*B gene, coding for an arabinosyltransferase participating in mycobacterial cell wall synthesis, with codon 306 being most frequently affected [14]. Furthermore, mutations in other parts of *emb*B (e.g. codon 406) [15] and upstream of *emb*A [15,16] and in *emb*C [16,17] are also involved in EMB resistance. Resistance to pyrazinamide (PZA) is known to be mediated by mutations occurring throughout the *pnc*A gene, encoding a pyrazinamidase [18]. Resistant strains lack pyrazinamidase activity which is essential for pro drug activation.

Since the frequency and combination of resistance mutations differs depending on the geographical setting in which the specific isolate is found [19,20], it is important to analyze *Mycobacterium tuberculosis* complex (MTBC) strains from different regions and to determine putative setting specific molecular markers. However, up to now data about the accuracy of molecular diagnostic methods in high-incidence settings, and especially in West Africa, is only sparely available. Therefore we carried out a population based study, involving MTBC strains from Sierra Leone, to determine the genetic basis of first line drug resistance and to compare results from molecular and conventional drug susceptibility testing.

## Methods

### Mycobacterial strains and growth conditions

**Figure 1:**
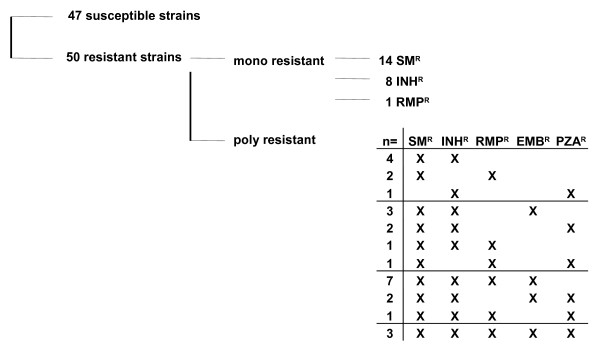
**Overview of the antibiotic resistance profiles of the strains analyzed.** A total of 97 *M. tuberculosis* and *M. africanum* strains from smear positive, previously treated patients from Sierra Leone was included in this study. Samples were collected in 2003 and 2004 in the Western Area and Kenema districts. Of the strains analyzed 74 were *M. tuberculosis* and 23 were *M. africanum* strains. Abbreviations: INH, isoniazid; RIF, rifampin; SM, streptomycin; EMB, ethambutol; PZA, pyrazinamide; R, resistance.

A total of 97 MTBC strains isolated from previously treated patients in Sierra Leone were included in this study. All smear positive cases registered for re-treatment (failure after at least 5 months, relapses or treatment after interruption) between March 2003 and June 2004 in the Western Area and Kenema districts in Sierra Leone were recruited. From the strains analyzed 50 were resistant to at least one of the following drugs INH, RIF, SM, EMB and PZA and 47 strains were fully susceptible (see Figure 1). From the panel of strains analyzed, 74 were *M. tuberculosis* and 23 were *M. africanum* strains. Primary isolation and cultivation was done at the Supranational Reference Laboratory in Borstel as described previously [21].

### Drug susceptibility testing

Drug susceptibility testing (DST) to first-line drugs INH (0.25 and 1.0 μg/ml), RIF (20.0 and 40.0 μg/ml), SM (4.0 and 8.0 μg/ml) and EMB (1.0 and 2.0 μg/ml) was performed in Borstel by using the proportion method on Löwenstein-Jensen (LJ) medium. In case of insufficient growth on LJ media, DST was done by applying the modified proportion method in the BACTEC 460 TB system according to the manufacturer`s instructions (Becton-Dickinson). Drug susceptibility testing to PZA (100 μg/ml) was performed by using the BACTEC™ Pyrazinamide (PZA) Drug Kit in the BACTEC 460 TB system according to the manufacturer`s instructions. Determination of minimal inhibitory concentrations (MICs) was done by applying the modified proportion method in the MGIT 960 TB system (test concentrations were 1.0, 0.5, 0.25, 0.125 and 0.063 μg/ml for RIF and SM, 100.0, 50.0, 25.0, 12.5 and 6.3 μg/ml for PZA).

### DNA Isolation, PCR and sequencing

DNA was isolated as described elsewhere [22] and amplified using the primers and conditions listed in Additional file 1. The PCR products were sequenced using an ABI 3130*xl* Genetic Analyzer (Applied Biosystems**,** CA, US) and the ABI BigDye Terminator kit v.1.1 according to the manufacturer’s instructions. The sequence data was analyzed using DNASTAR Lasergene version 8.0, with *M. tuberculosis* H37Rv DNA as reference sequence.

All strains were sequenced in the predominant resistance determining regions (RDR) of *kat*G (codon 315), *rpo*B (codon 507–533 according to *E. coli* numbering), *rrs* (nt. 1401–1402), *rps*L (complete gene), *gid*B (complete gene), *emb*B (codon 306) and *pnc*A (complete gene). Strains resistant to the respective drug but not carrying a mutation in the RDR were sequenced in the complete gene. Strains resistant to INH with no mutation in *kat*G were also sequenced in the promoter regions of *inh*A and *ahp*C. Similarly, we extended our sequencing effort to *emb*C and *emb*A for EMB resistant strains without any mutations in *emb*B.

## Results

In this study a total of 97 MTBC strains with known resistance patterns to first-line drugs from Sierra Leone (see Figure 1) were sequenced in genes previously described to be involved in resistance development.

**Figure 2:**
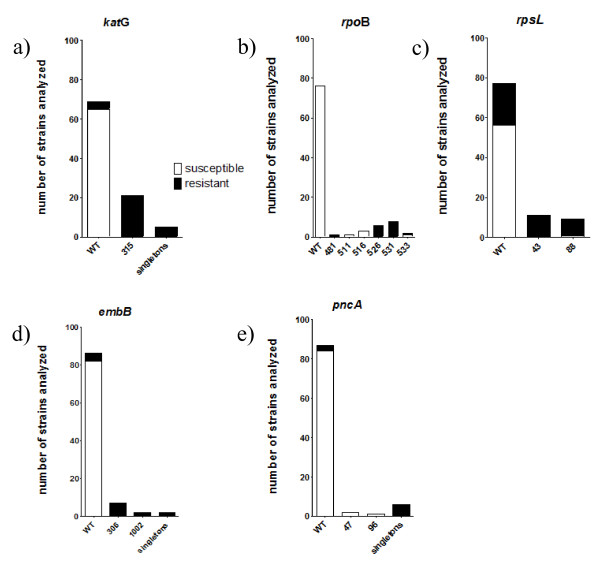
**Overview of mutations detected in all strains analyzed in a)*kat*G, b)*rpo*B, c)*rps*L, d)*emb*B and e)*pnc*A.** The height of the columns represent the number of strains analyzed. On the x-axis the different mutated codons are shown. The white areas of the columns represent the fraction of susceptible strains, whereas the black areas correspond to the number of resistant strains. Abbreviations: WT, wild type; singletons, various codons that are affected in one strain only.

The population structure of the strains was analyzed in a previous study [21]. Briefly, the 74 *M. tuberculosis* and 23 *M. africanum* strains belonged to eleven different genotypes. The population diversity was high with two *M. africanum* lineages (West African I, n = 6; West African II, n = 17) and nine *M. tuberculosis* lineages (Haarlem, n = 14; LAM, n = 15; EAI, n = 4; Beijing, n = 4; S-type, n = 4; X-type, n = 1; Cameroon, n = 4; Sierra Leone I, n = 7; Sierra Leone II, n = 10). To determine if certain mutations appear genotype specific, the occurrence of identified polymorphisms was correlated with the genotype of the respective strain. However, all mutations detected by sequencing analysis were found independently from their phylogenetic background (data not shown). A detailed summary of the sequencing data is provided in Table 1 and in Figure 2 (a-e).

**Table 1:** Mutations detected in all strains analyzed

**Gene**	**Mutation**	**Strains phenotypically susceptible to INH (n=)**	**Strains phenotypically resistant to INH (n=)**
*kat*G	wild type	65	4^(1)^
	Ala291Thr (gct/act); Ser315Thr (agc/acc)		1
	Trp300Cys (tgg/tgc)		1
	Thr302Arg (acg/agg)		1
	Ser315Thr (acg/acc)		21
	Asp329 (gac/ggac frameshift)		1
	Arg463Leu (cgg/ctg)^(2)^		2
	Gln471Arg (cag/cgg); Ser315Thr (agc/acc)		1
		**Strains phenotypically susceptible to RIF (n=)**	**Strains phenotypically resistant to RIF (n=)**
*rpo*B	wild type	76	
	Thr481Ala (acc/gcc)^(3)^		1
	Leu511Pro (ctg/ccg)^(4)^	1	
	Asp516Tyr (gac/tac)^(4)^	3	
	His526Tyr (cac/tac)		3
	His526Arg (cac/cgc)		3
	Ser531Leu (tcg/ttg)		8
	Leu533Pro (ctg/ccg)^(4)^	1	1
		**Strains phenotypically susceptible to SM (n=)**	**Strains phenotypically resistant to SM (n=)**
*rps*L	wild type	56	21
	Lys43Arg (aag/agg)		11
	Lys88Arg (aag/agg)	1	8
*gid*B	wild type	26 (2 with mutations in *rrs*/*rps*L)	15 (10 with mutations in *rps*L)
	Leu16Arg (ctt/cgt)	4	2
	Leu16Arg (ctt/cgt); Gln127stop (caa/taa); Ala161Ala (gcc/gcg)	1	
	Leu16Arg (ctt/cgt); Ala161Ala (gcc/gcg)	2	1 (Lys43Arg in *rps*L)
	Leu16Arg (ctt/cgt); Ala200Glu (gcg/gag)		2
	Leu16Arg (ctt/cgt); Ala205Ala (gca/gcg)		1 (Lys43Arg in *rps*L)
	Gly34Ala (ggg/gcg)		1
	Pro38 (ccc/cc frameshift)		1 (Lys88Arg in *rps*L)
	Val65Gly (gtc/ggc)		1
	Gly69Asp (ggt/gat)	1	2
	Gly71Arg (gga/aga)		1
	Val88Ala (gta/gca)		1
	Leu91Pro (cta/cca)		1
	Gly92Asp (gaa/gac)	1	
	Gly92Asp (gaa/gac); Ala205Ala (gca/gcg)		3 (Lys43Arg in *rps*L)
	Ser100Phe (tct/ttt)		1
	Val110Val (gtg/gtt); Ala205Ala (gca/gcg)	4	
	Ala138Val (gcg/gtg)		2
	Ala200Glu (gcg/gag) + Ala205Ala (gca/gcg)		1
	Ala205Ala (gca/gcg)	18	4 (3 with mutations in *rps*L)
		**Strains phenotypically susceptible to EMB (n=)**	**Strains phenotypically resistant to EMB (n=)**
*emb*B	wild type	82	4^(5)^
	Met306Ile (atg/ata)		6
	Met306Val (atg/gtg)		1
	Trp332Arg (tgg/cgg)		1
	Gln497Lys (cag/aag)^(3)^		1
	Gln1002Arg (cag/cgc)^(3)^		2
		**Strains phenotypically susceptible to PZA (n=)**	**Strains phenotypically resistant to PZA (n=)**
*pnc*A	wild type	84	3
	−11 (a/g)		1
	Ile5 (atc/atcc frameshift)		1
	Thr47Ala (acc/gcc)	2	
	Tyr64 (tat/tata frameshift)		1
	Lys96Glu (aag/gag)	1	
	Gln141 (cag/cag acggcgccag (insertion of 10 bp → frameshift)		1
	Ala146Glu (gcg/gag)		1
	Gly162Asp (ggt/gat)		1
	Leu172Pro (ctg/ccg)		1

Sequence analysis and drug susceptibility testing has been repeated for all strains showing discrepant results.

Abbreviations: INH, isoniazid; RIF, rifampin; SM, streptomycin; EMB, ethambutol; PZA, pyrazinamide.

^(1)^Those strains were additionally sequenced in the promoter region of *inh*A and *ahp*C. Two of them carried a mutation in *inh*A at position −15 (C → T) and one in *ahp*C at −57 (C → T).

^(2)^Arg463Leu is a phylogenetic SNP [23] and was excluded for further analysis.

^(3)^These mutations were identified by sequence analysis of the complete gene.

^(4)^These mutations confer low-level RIF resistance.

^(5)^These strains were sequenced in the complete *emb*CAB operon. One strain carried a mutation in *emb*C [Val981Leu (gtg/ctg)].

Among the INH resistant strains 71.9% (23/32) carried a mutation in *kat*G at codon 315. Out of these, 21 displayed a mutation in *kat*G only, while two strains showed mutations at *kat*G315 with additional mutations at codon 291 and codon 471, respectively. One strain each carried a mutation at codon 300, codon 302 and codon 329. Two resistant strains displayed a mutation at codon 463, which is a phylogenetic SNP [23] and was therefore excluded from further analysis. Four of the INH resistant strains had no mutation in *kat*G. However, sequence analysis of the intergenic regions of *inh*A and *ahp*C revealed polymorphisms in those areas. Two strains carried a mutation in *inh*A at position −15 and one strain in *ahp*C at −57. All of the 65 INH susceptible strains lacked mutations in *kat*G. Thus for detection of INH resistance, sequence analyses of *kat*G had a sensitivity and specificity of 86.7% and 100%, in the strains analyzed.

Among RIF resistant strains, 50% (8/16) carried a mutation in *rpo*B at codon 531. The second most frequent mutation was found at codon 526 (37.5%). One RIF resistant strain each showed a mutation at codon 481 and at codon 533, respectively. Out of 81 RIF susceptible strains 76 did not have any mutation in *rpo*B. The remaining five susceptible strains displayed mutations at codons 511 (n = 1), 516 (n = 3) and 533 (n = 1), respectively. Sequence analysis and drug susceptibility testing has been repeated for those five strains, confirming results of the first analyses. Determination of MICs revealed low-level RIF resistance (0.25-1.0 μg/ml) for those strains (see Table 2). Given that the strains showing low-level RIF resistance are assessed as susceptible by using standard DST, sequence analyses of *rpo*B had a sensitivity and specificity of 100% and 93.8% for detection of RIF resistance, in the strains analyzed.

**Table 2:** Determination of minimal inhibitory concentrations (MICs) of potential low-level resistant strains (to RIF, SM, PZA)

**strain**	**mutation**	**RIF MIC [μg/ml]**
4518/03	*rpo*B Asp516Tyr (gac/tac)	0.5
5472/03	*rpo*B Leu533Pro (ctg/ccg)	1.0
10011/03	*rpo*B Asp516Tyr (gac/tac)	0.5
3736/04	*rpo*B Leu511Pro (ctg/ccg)	0.5
6467/04	*rpo*B Asp516Tyr (gac/tac)	0.25
H37Rv control	wild type	0.25
**strain**	**mutation**	**SM MIC [μg/ml]**
6463/04	*rps*L Lys88Arg (aag/agg)	0.5
H37Rv control	wild type	0.5
**strain**	**mutation**	**PZA MIC [μg/ml]**
4724/03	*pnc*A Thr47Ala (acc/gcc)	25.0
4730/03	*pnc*A Thr47Ala (acc/gcc)	25.0
6467/04	*pnc*A Lys96Glu (aag/gag)	12.5
H37Rv control	wild type	12.5

To investigate the genetic basis of SM resistance, all strains were first sequenced in the *rrs* gene. As none of the resistant strains displayed a mutation in this gene, sequence analysis of *rps*L was performed. Among all SM resistant strains 27.5% (11/40) carried a mutation in *rps*L at codon 43 and 20% (8/40) showed a polymorphism at codon 88. The remainder of the phenotypically resistant strains (n = 21) did not carry a mutation in *rps*L. Among all SM susceptible strains (n = 57), one had the codon 88 mutation in *rps*L as well (confirmed when retested). Determination of SM MIC showed no elevated MIC for the respective strain compared to the H37Rv control (see Table 2). Taken together, these data resulted in a sensitivity and specificity of the DNA sequencing of *rps*L for detection of SM resistance of 48.8% and 98.2%, respectively.

Additionally all strains were sequenced in *gid*B. In this very polymorphic gene 16 different mutations have been found, which occurred alone or in combination (see Table 1). Noticeable is the high number of phylogenetic polymorphisms. The Leu16Arg (ctt/cgt) mutation was exclusively found in strains of the LAM genotype (n = 12). All strains belonging to the WA1, WA2 and Beijing genotypes displayed the Ala205Ala (gca/gcg) mutation (n = 27) and in all EAI strains a combination of the Val110Val (gtg/gtt) and Ala205Ala (gca/gcg) mutations was detected (n = 4). The role of mutations in *gid*B for resistance to SM needs to be further investigated.

Among all EMB resistant isolates 46.7% (7/15) carried a mutation in *emb*B at codon 306. One EMB resistant strain was found to have a mutation at codon 332, one at codon 497 and two strains carried a polymorphism at codon 1002. In four EMB resistant isolates no mutation in *emb*B was detected. Sequence analyses of *emb*C and *emb*A revealed a mutation in *emb*C [Val981Leu (gtg/ctg)] in one strain. All EMB susceptible strains (n = 82) had a wild-type *emb*B sequence. Thus for detection of EMB resistance, sequence analyses of *emb*B had a sensitivity and specificity of 73.3% and 100.0%, in the strains analyzed.

PZA resistant isolates showed a wide variety of changes, distributed throughout the entire length of the *pnc*A gene, including its promoter. Single nucleotide polymorphisms (SNPs) occurred in one strain each at position −11 bp, at codons 146, 162 and 172. In addition, insertions of single nucleotides leading to open reading frameshifts were detected at codons 5 and 64; an insertion of 10 bp after codon 141 led to PZA resistance in one strain. In three resistant isolates no mutation in *pnc*A was determined. Among all PZA susceptible strains (n = 87), 84 displayed the wild type sequence, whereas in three PZA susceptible strains mutations were detected at codon 47 (n = 2) and at codon 96 (n = 1), respectively. Sequence analysis and drug susceptibility testing has been repeated for strains showing discrepant results, however leading to unaltered findings. Determination of PZA-MICs (see Table 2) revealed slightly elevated MICs for the strains carrying the mutation at codon 47 (25.0 μg/ml) compared to the H37Rv control, but an unaltered MIC for the strain carrying the polymorphism at codon 96. These data resulted in a sensitivity and specificity of the DNA sequencing of *pnc*A for detection of PZA resistance of 70% and 96.6%, respectively.

A summary of all sequencing, DST, MIC and genotyping data is provided in Additional file 2.

## Discussion

In this study we carried out an in depth investigation of molecular resistance mechanisms by correlating particular genomic variants with phenotypic resistance in clinical isolates from a high-incidence setting in West Africa. For INH and RIF there is a close correlation between data from molecular and phenotypic resistance testing for resistance determination in the strains analyzed. Sensitivity and specificity of sequencing of *kat*G for detection of drug resistance were 86.7% and 100% and for sequencing of *rpo*B 100% and 93.8%, respectively.

Overall, the correlation between molecular and phenotypic resistance testing for the determination of SM, EMB and PZA resistance was lower. Although specificities of sequencing of *rps*L, *emb*B and *pnc*A were high (96-100%), sensitivities were lower (48-73%) due to so far unknown resistance mechanisms.

However, while our results in principle support molecular resistance testing, the finding that especially in *rpo*B and also in *pnc*A particular mutations are not linked to high-level resistance is challenging and demonstrates that careful interpretation of molecular resistance assays is mandatory. Therefore, studies targeting new resistance mechanisms should include valid phenotypic resistance data and, to our opinion, a comprehensive database on genetic variations in resistance genes and the correlation with phenotypic resistance is necessary. Furthermore, the level of resistance mediated by particular mutations and the clinical consequences need to be thoroughly investigated. In addition, especially variations in *gid*B appear to be phylogenetically restricted rather than being involved in drug resistance development.

In our study the most frequent mutation among INH resistant strains has been detected in *kat*G at codon 315. This SNP has been observed in numerous prior studies [24,25] and has clearly been correlated with INH resistance by loss of catalase activity. In two strains, in addition to variations at *kat*G315, mutations at codon 291 and 471 were detected. However neither mutation has been described in the literature before and the *kat*G315 mutation therefore represents the likely mechanism for INH resistance in these strains. The mutation at codon 300 observed in one strain in our study has been previously reported by Richardson and co-workers [26], where loss of this mutation has resulted in reversion of INH resistance in a previously drug resistant strain. The mutation at codon 302 as well as the insertion at codon 329 has not been described previously. Since they are restricted to INH resistant strains in our highly diverse MTBC collection, they represent potential new INH resistance mechanisms. Experimental evidence is required to validate this hypothesis.

Of the four INH resistant strains that displayed a wild-type *kat*G sequence, three had mutations in the promoter regions of *inh*A and *ahp*C. The *inh*A mutation has previously been described in the literature [24] as being the most common variation in the *inh*A promoter region related to INH resistance. Mutations in *ahp*C have been found before, however to our knowledge not at this position. In one of the resistant strains no mutation was found in neither the complete *kat*G gene nor in *inh*A or in *ahp*C. This result suggests a so far unknown resistance mechanism as being responsible for INH resistance of this strain.

Mutations in *rpo*B at codons 526 and 531 occur most frequently in the RIF resistant strains analyzed. Those SNPs are located in the RRDR and are well known for mediating resistance [27,28]. The mutation at codon 481, which only occurs in one RIF resistant isolate, has to our knowledge not been described previously.

The mutations at codon 511 (Leu → Pro), 516 (Asp → Tyr) and 533 (Leu → Pro) conferred low-level resistance in agreement with previous studies [29,30]. It has been shown that various substitutions in the same codon lead to different levels of resistance. For example mutations at codon 516 can confer either low- or high-level resistance depending on the amino acid change [30]. Furthermore, the phenomenon of RIF low-level resistance has only recently been described in a work by Van Deun and colleagues [31], where mutations at codon 511, 516 and 533 have been found in strains tested susceptible by the radiometric Bactec 460 TB and Bactec 960 MGIT methods. Our data confirm the existence of low-level RIF resistance mediated by specific mutations in *rpo*B that is not detected by standard drug susceptibility testing methods. However, MIC values, especially for the mutations at codon 516 and 533, are even lower (0.5-1.0 μg/ml) than have been described in the literature. This fact may be due to the presence of further mutations in the operon or in other regions of the genome.

In a recent study [32] the therapeutic challenge of low-level RIF resistance has been addressed and may, according to the authors, be overcome by the application of higher RIF doses (20 mg/kg) in treatment regimens. However, the clinical relevance and interpretation of these data is still not fully understood and needs further investigation in animal treatment models or clinical trials.

Despite these discordant findings, we found a good correlation between the results from molecular and phenotypic testing for INH and RIF, as has been observed in another study [33]. In fact, the strains analyzed in this study predominantly harbour well described mutations which allows for the application of standard sequencing protocols or commercial line probe assays.

The analysis of SM resistance mechanisms revealed an interesting observation. None of the SM resistant strains carried a mutation in the *rrs* gene, although those mutations have been described as main resistance mechanisms that confer high-level SM resistance [12]. Instead, the SM resistant strains in our study population carry mutations in *rps*L at codon 43 or 88 or at various codons in the *gid*B gene. The two mutations in *rps*L have been described previously to confer high-level SM resistance [28,34]. Polymorphisms in *gid*B were reported to confer a lower level of SM resistance [13]. However, due to a number of phylogenetic polymorphisms in *gid*B, cautious interpretation of sequencing data is mandatory. Leu16Arg (ctt/cgt) has been described previously as phylogenetic marker for the LAM genotype [35], which could be confirmed in this study. Additionally, a synonymous SNP at codon Ala205Ala (gca/gcg) was identified as being specific for the WA1, WA2 and Beijing genotypes, as well as a combination of Ala205Ala (gca/gcg) and Val110Val (gtg/gtt) was determined as phylogenetically specific for strains belonging to the EAI genotype. These mutations in *gid*B occurred both in SM susceptible and resistant strains, affirming their role as phylogentic SNPs rather than markers for SM resistance. Polymorphisms in *gid*B probably playing a role in SM resistance, as they occur exclusively in SM resistant strains and do not coincide with mutations in *rps*L, were detected throughout the complete gene (codons 34, 65, 71, 88, 91, 100, 138, 200). However, the actual importance of these SNPs for SM resistance needs to be investigated in further studies.

Reasons for the absence of *rrs* mutations in the strains analyzed and the shift to mutations in *rps*L and *gid*B are mainly unclear, but are in line with previous studies reporting a disequilibrium in the distribution of resistance conferring mutations in different geographical areas or among strains of different genotypes [36-38]. Our findings confirm that the performance of molecular assays that only target particular mutations can be influenced by the differential prevalence of particular mutations in a given geographical area. Therefore, strain diversity needs to be considered and investigated before the new implementation of molecular assays in a study region.

Among EMB resistant isolates, the most frequent mutation affected codon 306 (Met/Ile) of the *emb*B gene. This mutation has been described in various studies as the main mutation mediating resistance to EMB [14,39]. The mutation at codon 497 has also been previously described in clinical isolates [40]. Moreover, both mutations have been shown to confer resistance by transfer in a wild type genetic background using allelic exchange experiments [41]. However, the authors conclude that single mutations only modestly increase resistance to EMB and additional so far unknown mutations are necessary to cause high-level resistance.

The mutations at codon 332 and 1002 determined here have not been described before. The impact of these changes has to be investigated in further studies. In four resistant strains no mutations were detected in the *emb*B region analyzed. Additional analysis of the *emb*C and *emb*A genes revealed a mutation in *emb*C [Val981Leu (gtg/ctg)] in one strain, belonging to the Haarlem genotype. However, this mutation has been described earlier as being specific for the Haarlem genotype and is not associated with resistance to EMB [16]. As mentioned above, other so far unknown resistance mediating mechanisms are probably responsible for the resistance phenotype in these four strains.

Mutations or insertions in the *pnc*A gene are known to mediate PZA resistance [42,43], as observed in our study. No hotspot region has been determined, since polymorphisms occur throughout the complete gene. However, according to our data some specific mutations do obviously not mediate resistance that is detectable by applying standard critical concentrations. In the panel of strains analyzed, two susceptible strains carry a SNP at codon 47 and one displays a mutation at codon 96. PZA-MIC determination for these strains revealed slightly elevated values for the strains carrying the mutation at codon 47 (25.0 μg/ml) compared to the H37Rv control. In a recent study it has been shown that the site of the mutation is leading to varying efficiencies of the mutated pyrazinamidase mediating a wide range of resistance levels from low to high [44]. As the mutation at codon 47 has previously been described by Juréen and co-workers [42] in PZA resistant strains, further investigations are necessary to determine if additional mutations in other parts of the genome might be responsible for the observed low-level resistance in the strains analyzed in this study. Out of all PZA resistant strains three carried the *pnc*A wild type sequence. This indicates that further mutations in as yet unidentified genes are also important for mediating PZA resistance.

## Conclusions

Although resistance mechanisms to INH and RIF are well understood, unknown resistance determining regions and resistance mediating mechanisms appear to play an important role for SM, EMB and PZA, where we observed a relatively low sensitivity for detection of resistance by analysis of common genes. Therefore, it is essential to gather information on further mechanisms leading to drug resistant MTBC strains. For the design and implementation of molecular resistance assays it is fundamental to consider strain diversity with respect to resistance mutations in a given geographical setting. Finally, it should be noted that not all variations in well described resistance genes are related to the development of high-level resistance, a finding arguing for a very careful interpretation of molecular resistance assays.

## Competing interests

The authors declare that they have no competing interests.

## Authors’ contributions

SF: Conception and design of the study, acquisition, analysis and interpretation of data, drafting and revising of the article, given final approval to this version to be published. BO: Conception and design of the study, revising of the article, given final approval to this version to be published. AGG: Conception and design of the study, revising of the article, given final approval to this version to be published. FD: Conception and design of the study, revising of the article, given final approval to this version to be published. ER: Conception and design of the study, interpretation of data, revising of the article, given final approval to this version to be published. SR-G: Conception and design of the study, interpretation of data, revising of the article, given final approval to this version to be published. SN: Conception and design of the study, interpretation of data, drafting and revising of the article, given final approval to this version to be published. All authors read and approved the final manuscript.

## Supplementary Material

Additional file 1 PCR primers and conditions used for amplification and sequencing. In this table a summary of all primers, including oligonucleotide sequences, used in this study for both DNA amplification and sequencing is given. Click here for file

Additional file 2 Summary of all sequencing, DST, MIC and genotyping data. This table summarizes all data generated in this study. It comprises sequencing, DST (drug susceptibility testing) and MIC (minimal inhibitory concentration) testing results as well as all genotyping data. Click here for file
