# Supplementary material for: Sequence analysis for detection of first-line drug resistance in Mycobacterium tuberculosis strains from a high-incidence setting
Source: BMC Microbiol. 2012 May 30;12:90. doi: 10.1186/1471-2180-12-90 (PMC3404943; doi:10.1186/1471-2180-12-90)
Supplement: Additional file 1 — PCR primers and conditions used for amplification and sequencing. In this table a summary of all primers, including oligonucleotide sequences, used in this study for both DNA amplification and sequencing is given. [file 1471-2180-12-90-S1.docx]

**Additional Table 1. PCR primers and conditions used for amplification and sequencing.**

| **Primer (position relative to gene start)** | **Primer sequence (5`→3`)** | **Size (bp)** | **D (s)^a^** | **A (°C, s)** | **E (s)** |
| --- | --- | --- | --- | --- | --- |
| *kat*G_5`(-182) | CGC CGA TGT CGA CTG TGC TGT T | 690 | 30 | 65.0, 30 | 45 |
| *kat*G_3`(+508) | TGC CGG CGA AAA CAA TCA GGT C |  |  |  |  |
| *kat*G_5`(+331) | GGC ACC TAC CGC ATC CAC | 1511 | 30 | 55.0, 30 | 90 |
| *kat*G_3`(+1842) | CGC CTT GTC GAG CAG CAT |  |  |  |  |
| *kat*G_5`(+1592) | CGG CGC CGG GGA ACA TCA AA | 751 | 30 | 60.0, 30 | 45 |
| *kat*G_3`(+2343) | CCA TGC ACG CGG GGT CTG |  |  |  |  |
| *inh*A-Primer TB 92 | CCT CGC TGC CCA GAA AGG GA | 250 | 30 | 61.0, 30 | 30 |
| *inh*A-Primer TB 93 | ATC CCC CGG TTT CCT CCG GT |  |  |  |  |
| *ahp*C-Primer TB 90 | CCG ATG AGA GCG GTG AGC TG | 250 | 30 | 61.0, 30 | 30 |
| *ahp*C-Primer TB 91 | ACC ACT GCT TTG CCG CCA CC |  |  |  |  |
| *rpo*B_5`(-95) | GGC CGA AAC CGA CAA AAT | 1303 | 30 | 53.0, 30 | 90 |
| *rpo*B_5`(+338)^b^ | TCG ACG AGT GCA AAG ACA AG |  |  |  |  |
| *rpo*B_3`(+1208) | ACG TCC TGG GTG GTC ATC C |  |  |  |  |
| *rpo*B_5`(+1087) | GAC ATC GAC CAC TTC GGC AA | 2532 | 30 | 57.0, 30 | 150 |
| *rpo*B_5`(+1785)^b^ | CGC CAA CCG TGC CCT CAT |  |  |  |  |
| *rpo*B_5`(+2365)^b^ | GAG GTT CGC GAC GGG GAC AT |  |  |  |  |
| *rpo*B_3`(+3619) | TCC TCC GCG GTA GCA AGA C |  |  |  |  |
| *rrs*_5´(+1202) | CTT ATG TCC AGG GCT TCA | 363 | 30 | 50.0, 30 | 30 |
| *rrs*_3´(+1565) | CAG TTG GGG CGT TTT C |  |  |  |  |
| *rrs*_5`(-181) | CGA AGC GGG CGG AAA CAA | 1746 | 30 | 50.0, 30 | 100 |
| *rrs*_5`(+407)^b^ | GGG ATG ACG GCC TTC GGG TTG T |  |  |  |  |
| *rrs*_3´(+1565) | CAG TTG GGG CGT TTT C |  |  |  |  |
| *rps*L_5`(-112) | ATG AGA CGA ATC GAG TTT GAG | 530 | 30 | 55.0, 30 | 45 |
| *rps*L_3`(+418) | GCT CAA GCG CAC CAT AAA CAA |  |  |  |  |
| *gid*B_5`(-152) | CGC CGA GTC GTT GTG CT | 886 | 30 | 55.0, 30 | 60 |
| *gid*B_3`(+734) | AGC CTG GCC CGA CCT TA |  |  |  |  |
| Rv3795anewF | CTG GGG ATC GGT GGA GCA GTA | 969 | 30 | 65.0, 30 | 60 |
| Rv3795anewR | GCG TCG GTC AGG GTG AAG G |  |  |  |  |
| Rv3795bF | TGG ACG GGC GGG GCT CAA T | 1305 | 30 | 65.0, 30 | 90 |
| Rv3795bcR | GCA AAC AGG GCG AAA AAG A |  |  |  |  |
| Rv3795cF | TCC TGG CGG CGT TAT TCT T | 1652 | 30 | 65.0, 30 | 100 |
| Rv3795cS1^b^ | TGG ACG GCG ATT CGG GTT CT |  |  |  |  |
| Rv3795cS2^b^ | GGA CTG GGC GGT CGG TTT G |  |  |  |  |
| Rv3795cR | CAA CCG GGG TGA TGA TGG C |  |  |  |  |
| embIR-F^c^ | CTG GTG GTC GCG GTG ATC AT | 907 | 30 | 64.0, 45 | 60 |
| embIR-newR | ACG GTC GCT GGC AGG GGA AGT T |  |  |  |  |
| embIR-newF | CGC CTA TGA CCC GAA CCT GAG | 787 | 30 | 65.0, 45 | 60 |
| embIR-R^c^ | AAT TGG CGT CCT TGC CTT |  |  |  |  |
| embA1-F^c^ | GTG ACT CGC AGC GGG CTG TG | 1223 | 30 | 68.0, 30 | 90 |
| embA1-R^c^ | CGG TGA ACA CAG CGA CCC GG |  |  |  |  |
| embA2-F^c^ | TGG ACC GGC TCA GCA GGG G | 1500 | 30 | 67.0, 30 | 90 |
| embA2-R^c^ | TCA GGT TGG CCT TGG CGG TG |  |  |  |  |
| embC1-F^c^ | CCC AAC CAG CCC AAT GTT C | 890 | 40 | 64.5, 30 | 60 |
| embC1-R^c^ | GGC GGT GTC CAG GAT GTG |  |  |  |  |
| embC2-F^c^ | GCT GCA CAT CCT GGA CAC | 914 | 40 | 60.0, 30 | 60 |
| embC2-R^c^ | ACG ACA TTG CCA CCG ATA C |  |  |  |  |
| embC3-F^c^ | GTA TCG GTG GCA ATG TCG T | 1176 | 40 | 60.0, 30 | 60 |
| embC3-R^c^ | CGG GAT GGC GGA CAG TGG T |  |  |  |  |
| embC4-F^c^ | ACC ACT GTC CGC CAT CCC G | 635 | 30 | 67.0, 30 | 45 |
| embC4-R^c^ | GAC GAC GGC TGC TAG GCG TG |  |  |  |  |
| *pnc*A1 | GCT GGT CAT GTT CGC GAT CG | 664 | 30 | 60.0, 30 | 45 |
| *pnc*A2 | CAG GAG CTG CAA ACC AAC TCG |  |  |  |  |

^a^ D, length of denaturation at 95°C; A, primer annealing conditions (temperature in °C and length in s); E, length of extension at 72°C. All PCRs were 35 cycles, were preceded by an initial denaturation step at 95°C for 15 min, and included a final extension step at 72°C for 5 min.

^b^ sequencing primer

^c^ see Ramaswamy et al. [15]
